# He Comes Back

**Published:** 1888-11

**Authors:** 


HE COMES BACK.—An old and highly esteemed subscriber writes us: "Some years ago I stopped your journal thinking I could do better elsewhere, but after trying a number of other journals I go back to the OLD RECORD."

We have received from time to time, many such letters. Should this number fall into the hands of any who desire a change, we hope they will be thereby influenced to stay with us, and should it fall into the hands of any who have quit us for journals no better, and,perhaps, not so good, that they too will come back to the old fold.

We are glad to announce that the great majority of our subscribers are true to us. Many having been on our list since the issue of our first number in 1870. And oft times we receive from them words of kindness and encouragement, and warm expressions of their appreciation of their Home Journal and of our labors in behalf of the medical literature of the South.

